# (*E*)-*N*-Benzyl-2-cyano-3-phenyl­acryl­amide

**DOI:** 10.1107/S1600536810049548

**Published:** 2010-12-04

**Authors:** Tai-Ran Kang, Lian-Mei Chen

**Affiliations:** a College of Chemistry and Chemical Engineering, China West Normal University, Nanchong 637002, People’s Republic of China

## Abstract

In the title compound, C_17_H_14_N_2_O, the *N*-benzyl­formamide and phenyl groups are located on the opposite sides of the C=C bond, showing an *E* configuration; the terminal phenyl rings are twisted to each other at a dihedral angle of 63.61 (7)°. Inter­molecular classical N—H⋯N and weak C—H⋯O hydrogen bonds occur in the crystal structure.

## Related literature

For the use of malononitrile-containing compounds as building blocks in syntheses, see: Lee *et al.* (2002 ▶); Rajan *et al.* (2001 ▶); Yingyongnarongkul *et al.* (2006 ▶). For a related structure, see: Kang & Chen (2009 ▶).
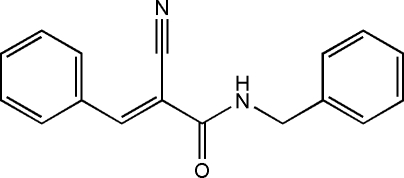


## Experimental

### 

#### Crystal data

C_17_H_14_N_2_O
*M* *_r_* = 262.30
Triclinic, 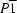

*a* = 5.8956 (3) Å
*b* = 9.9224 (5) Å
*c* = 12.1400 (7) Å
α = 94.508 (5)°
β = 99.544 (4)°
γ = 98.895 (4)°
*V* = 687.95 (6) Å^3^
*Z* = 2
Cu *K*α radiation
μ = 0.64 mm^−1^
*T* = 291 K
0.36 × 0.35 × 0.30 mm

#### Data collection

Oxford Diffraction Xcalibur Sapphire3 Gemini ultra diffractometer
Absorption correction: multi-scan (*CrysAlis PRO*; Oxford Diffraction, 2009 ▶) *T*_min_ = 0.803, *T*_max_ = 0.832
5416 measured reflections
2407 independent reflections
2202 reflections with *I* > 2σ(*I*)
*R*_int_ = 0.018

#### Refinement

*R*[*F* ^2^ > 2σ(*F* ^2^)] = 0.036
*wR*(*F* ^2^) = 0.096
*S* = 1.05
2407 reflections
185 parameters
2 restraints
H atoms treated by a mixture of independent and constrained refinement
Δρ_max_ = 0.14 e Å^−3^
Δρ_min_ = −0.12 e Å^−3^

### 

Data collection: *CrysAlis CCD* (Oxford Diffraction, 2008 ▶); cell refinement: *CrysAlis RED* (Oxford Diffraction, 2008 ▶); data reduction: *CrysAlis RED*; program(s) used to solve structure: *SHELXS97* (Sheldrick, 2008 ▶); program(s) used to refine structure: *SHELXL97* (Sheldrick, 2008 ▶); molecular graphics: *ORTEP-3* (Farrugia, 1997 ▶); software used to prepare material for publication: *SHELXL97*.

## Supplementary Material

Crystal structure: contains datablocks global, I. DOI: 10.1107/S1600536810049548/xu5098sup1.cif

Structure factors: contains datablocks I. DOI: 10.1107/S1600536810049548/xu5098Isup2.hkl

Additional supplementary materials:  crystallographic information; 3D view; checkCIF report

## Figures and Tables

**Table 1:** Hydrogen-bond geometry (Å, °)

*D*—H⋯*A*	*D*—H	H⋯*A*	*D*⋯*A*	*D*—H⋯*A*
N2—H4⋯N1^i^	0.88 (1)	2.24 (1)	3.0687 (14)	157 (1)
C3—H3⋯O1^ii^	0.93	2.36	3.2672 (16)	164

Symmetry codes: (i) 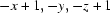; (ii) 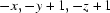.

**Fig. 1.**
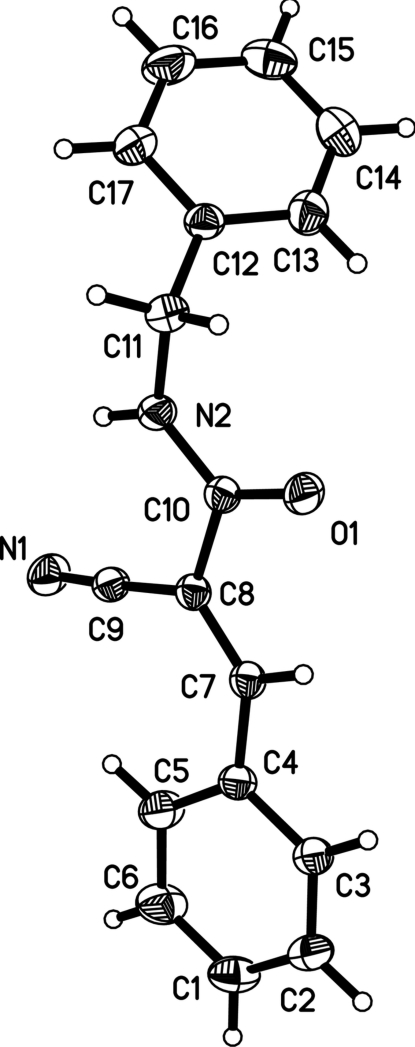
The molecular structure of (I) with 30% probability displacement ellipsoids (arbitrary spheres for H atoms).

## supplementary crystallographic information

### Comment

The phenylacrylamide derivatives have broad application for the preparation of heterocyclic ring compounds. The phenylacrylamide derivatives was studied extensively, Rajan (Rajan *et al.*,2001) synthesized a series of phenylacrylamide derivatives and evaluated their antioxidant properties as lipid peroxidation inhibitors. Some phenylacrylamide derivatives and analogues were synthesized and studied antibacterial activity against S. aureus (Yingyongnarongkul *et al.*,2006). Some phenylacrylamide derivatives were synthesized for the purpose of simplifying the structure of *L*-chicoric acid as new HIV-1 integrase inhibitors (Lee *et al.*, 2002).As an extension of this research, we report the synthesis and the crystal structure of the title compound (I), namely, (*E*)—*N*-benzyl-2-cyano-3-phenylacrylamide.

The molecular structure of (I) is shown in Fig. 1. Bond lengths and angles in (I) are normal. The dihedral angle between the C1—C6 and C12—C17 benzene planes is 63.62 (5)°. The crystal packing is stabilized by N—H···N an C—H···O hydrogen bonding (Table 1).

### Experimental

*N*-Benzyl-2-cyanoacetamide (0.258 g, 2 mmol) and benzaldehyde (0.212 g, 2 mmol) were dissolved in 2-propanol (2 ml). To the solution was added piperidine (0.017 g, 0.2 mmol),the solution was stirred for 24 h at 273 K and the solution was filtered to obtain a solid. Recrystallization from hot ethanol afforded the pure compound. Single crystals of (I) suitable for X-ray analysis were obtained by slow evaporation ethanol solvent.

### Refinement

Imino H atom was located in a difference Fourier map and refined isotropically. Other H atoms were placed in calculated positions, with C—H = 0.93–0.97 Å, and refined using a riding model, with *U*_iso_(H) = 1.2*U*_eq_(C).

### Crystal data

**Table:**

C_17_H_14_N_2_O	*Z* = 2
*M**_r_* = 262.30	*F*(000) = 276
Triclinic, *P*1	*D*_x_ = 1.266 Mg m^−^^3^
Hall symbol: -P 1	Cu *K*α radiation, λ = 1.54184 Å
*a* = 5.8956 (3) Å	Cell parameters from 3956 reflections
*b* = 9.9224 (5) Å	θ = 4.5–72.2°
*c* = 12.1400 (7) Å	µ = 0.64 mm^−^^1^
α = 94.508 (5)°	*T* = 291 K
β = 99.544 (4)°	Block, yellow
γ = 98.895 (4)°	0.36 × 0.35 × 0.30 mm
*V* = 687.95 (6) Å^3^	

### Data collection

**Table:**

Oxford Diffraction Xcalibur Sapphire3 Gemini ultra diffractometer	2407 independent reflections
Radiation source: Enhance Ultra (Cu) X-ray Source	2202 reflections with *I* > 2σ(*I*)
mirror	*R*_int_ = 0.018
Detector resolution: 7.9575 pixels mm^-1^	θ_max_ = 67.1°, θ_min_ = 4.5°
ω scans	*h* = −6→7
Absorption correction: multi-scan (*CrysAlis PRO*; Oxford Diffraction, 2009)	*k* = −11→11
*T*_min_ = 0.803, *T*_max_ = 0.832	*l* = −14→14
5416 measured reflections	

### Refinement

**Table:**

Refinement on *F*^2^	Primary atom site location: structure-invariant direct methods
Least-squares matrix: full	Secondary atom site location: difference Fourier map
*R*[*F*^2^ > 2σ(*F*^2^)] = 0.036	Hydrogen site location: inferred from neighbouring sites
*wR*(*F*^2^) = 0.096	H atoms treated by a mixture of independent and constrained refinement
*S* = 1.05	*w* = 1/[σ^2^(*F*_o_^2^) + (0.0452*P*)^2^ + 0.101*P*] where *P* = (*F*_o_^2^ + 2*F*_c_^2^)/3
2407 reflections	(Δ/σ)_max_ < 0.001
185 parameters	Δρ_max_ = 0.14 e Å^−^^3^
2 restraints	Δρ_min_ = −0.12 e Å^−^^3^

### Special details

**Table:**

Geometry. All e.s.d.'s (except the e.s.d. in the dihedral angle between two l.s. planes) are estimated using the full covariance matrix. The cell e.s.d.'s are taken into account individually in the estimation of e.s.d.'s in distances, angles and torsion angles; correlations between e.s.d.'s in cell parameters are only used when they are defined by crystal symmetry. An approximate (isotropic) treatment of cell e.s.d.'s is used for estimating e.s.d.'s involving l.s. planes.
Refinement. Refinement of *F*^2^ against ALL reflections. The weighted *R*-factor *wR* and goodness of fit *S* are based on *F*^2^, conventional *R*-factors *R* are based on *F*, with *F* set to zero for negative *F*^2^. The threshold expression of *F*^2^ > σ(*F*^2^) is used only for calculating *R*-factors(gt) *etc*. and is not relevant to the choice of reflections for refinement. *R*-factors based on *F*^2^ are statistically about twice as large as those based on *F*, and *R*- factors based on ALL data will be even larger.

### Fractional atomic coordinates and isotropic or equivalent isotropic displacement parameters (Å^2^)

**Table:**

	*x*	*y*	*z*	*U*_iso_*/*U*_eq_	
O1	0.28178 (15)	0.43568 (9)	0.56771 (8)	0.0523 (3)	
N2	0.53117 (17)	0.28418 (10)	0.58336 (8)	0.0424 (3)	
C4	−0.2206 (2)	0.19407 (12)	0.31358 (9)	0.0398 (3)	
C12	0.7670 (2)	0.35036 (12)	0.77490 (10)	0.0413 (3)	
N1	0.2529 (2)	−0.02233 (11)	0.42829 (10)	0.0556 (3)	
C8	0.1591 (2)	0.22182 (11)	0.45635 (9)	0.0371 (3)	
C3	−0.3994 (2)	0.26728 (13)	0.27993 (11)	0.0471 (3)	
H3	−0.3904	0.3563	0.3128	0.056*	
C9	0.20860 (19)	0.08566 (12)	0.43834 (10)	0.0405 (3)	
C7	−0.0298 (2)	0.26411 (11)	0.40119 (10)	0.0389 (3)	
H7	−0.0409	0.3545	0.4229	0.047*	
C11	0.7242 (2)	0.37901 (13)	0.65408 (11)	0.0458 (3)	
H11A	0.6918	0.4717	0.6509	0.055*	
H11B	0.8653	0.3745	0.6237	0.055*	
C10	0.32978 (19)	0.32315 (11)	0.54175 (9)	0.0375 (3)	
C2	−0.5896 (2)	0.20970 (15)	0.19856 (12)	0.0570 (4)	
H2	−0.7080	0.2596	0.1776	0.068*	
C17	0.9529 (2)	0.28819 (16)	0.81647 (12)	0.0576 (4)	
H17	1.0518	0.2634	0.7691	0.069*	
C15	0.8499 (3)	0.29793 (18)	0.99859 (13)	0.0689 (4)	
H15	0.8778	0.2804	1.0733	0.083*	
C13	0.6229 (2)	0.38575 (14)	0.84780 (12)	0.0536 (3)	
H13	0.4967	0.4276	0.8216	0.064*	
C5	−0.2386 (3)	0.06160 (14)	0.26135 (12)	0.0579 (4)	
H5	−0.1209	0.0109	0.2813	0.069*	
C6	−0.4295 (3)	0.00533 (15)	0.18032 (13)	0.0666 (4)	
H6	−0.4404	−0.0835	0.1467	0.080*	
C14	0.6643 (3)	0.35969 (16)	0.95881 (13)	0.0646 (4)	
H14	0.5661	0.3840	1.0067	0.078*	
C1	−0.6041 (3)	0.07908 (15)	0.14864 (12)	0.0613 (4)	
H1	−0.7318	0.0405	0.0935	0.074*	
C16	0.9936 (3)	0.26243 (19)	0.92760 (14)	0.0727 (5)	
H16	1.1195	0.2206	0.9543	0.087*	
H4	0.556 (2)	0.2006 (13)	0.5647 (12)	0.054 (4)*	

### Atomic displacement parameters (Å^2^)

**Table:**

	*U*^11^	*U*^22^	*U*^33^	*U*^12^	*U*^13^	*U*^23^
O1	0.0492 (5)	0.0412 (5)	0.0618 (6)	0.0158 (4)	−0.0034 (4)	−0.0101 (4)
N2	0.0437 (6)	0.0402 (5)	0.0415 (6)	0.0134 (4)	−0.0015 (4)	−0.0002 (4)
C4	0.0431 (6)	0.0387 (6)	0.0364 (6)	0.0082 (5)	0.0028 (5)	0.0033 (5)
C12	0.0383 (6)	0.0394 (6)	0.0423 (6)	0.0043 (5)	0.0002 (5)	0.0003 (5)
N1	0.0566 (7)	0.0418 (6)	0.0664 (7)	0.0175 (5)	−0.0001 (5)	0.0008 (5)
C8	0.0409 (6)	0.0347 (5)	0.0360 (6)	0.0089 (4)	0.0059 (5)	0.0023 (4)
C3	0.0502 (7)	0.0461 (7)	0.0434 (7)	0.0138 (5)	0.0008 (5)	0.0012 (5)
C9	0.0406 (6)	0.0386 (6)	0.0406 (6)	0.0097 (5)	0.0006 (5)	0.0016 (5)
C7	0.0436 (6)	0.0345 (6)	0.0388 (6)	0.0098 (5)	0.0061 (5)	0.0016 (5)
C11	0.0398 (6)	0.0491 (7)	0.0459 (7)	0.0060 (5)	0.0021 (5)	0.0051 (5)
C10	0.0405 (6)	0.0370 (6)	0.0355 (6)	0.0100 (5)	0.0059 (5)	0.0033 (4)
C2	0.0504 (8)	0.0660 (9)	0.0508 (8)	0.0160 (6)	−0.0066 (6)	0.0042 (6)
C17	0.0460 (7)	0.0747 (9)	0.0524 (8)	0.0190 (6)	0.0018 (6)	0.0059 (7)
C15	0.0760 (10)	0.0812 (11)	0.0422 (8)	0.0034 (8)	−0.0028 (7)	0.0101 (7)
C13	0.0560 (8)	0.0536 (8)	0.0543 (8)	0.0179 (6)	0.0109 (6)	0.0049 (6)
C5	0.0632 (8)	0.0441 (7)	0.0597 (8)	0.0163 (6)	−0.0101 (7)	−0.0042 (6)
C6	0.0784 (10)	0.0460 (8)	0.0625 (9)	0.0067 (7)	−0.0133 (8)	−0.0094 (7)
C14	0.0777 (10)	0.0672 (9)	0.0505 (8)	0.0113 (8)	0.0197 (7)	0.0003 (7)
C1	0.0586 (8)	0.0619 (9)	0.0514 (8)	−0.0007 (7)	−0.0119 (6)	0.0010 (7)
C16	0.0598 (9)	0.0964 (13)	0.0599 (9)	0.0238 (8)	−0.0093 (7)	0.0186 (9)

### Geometric parameters (Å, °)

**Table:**

O1—C10	1.2240 (13)	C11—H11B	0.9700
N2—C10	1.3372 (14)	C2—C1	1.371 (2)
N2—C11	1.4612 (15)	C2—H2	0.9300
N2—H4	0.884 (12)	C17—C16	1.382 (2)
C4—C5	1.3945 (17)	C17—H17	0.9300
C4—C3	1.3954 (16)	C15—C16	1.368 (2)
C4—C7	1.4574 (16)	C15—C14	1.373 (2)
C12—C17	1.3824 (17)	C15—H15	0.9300
C12—C13	1.3862 (18)	C13—C14	1.381 (2)
C12—C11	1.5036 (17)	C13—H13	0.9300
N1—C9	1.1435 (15)	C5—C6	1.3781 (19)
C8—C7	1.3443 (16)	C5—H5	0.9300
C8—C9	1.4334 (15)	C6—C1	1.374 (2)
C8—C10	1.5084 (16)	C6—H6	0.9300
C3—C2	1.3803 (18)	C14—H14	0.9300
C3—H3	0.9300	C1—H1	0.9300
C7—H7	0.9300	C16—H16	0.9300
C11—H11A	0.9700		
			
C10—N2—C11	122.18 (10)	C1—C2—C3	120.08 (13)
C10—N2—H4	120.6 (9)	C1—C2—H2	120.0
C11—N2—H4	117.1 (9)	C3—C2—H2	120.0
C5—C4—C3	117.89 (11)	C16—C17—C12	120.79 (14)
C5—C4—C7	125.69 (11)	C16—C17—H17	119.6
C3—C4—C7	116.41 (10)	C12—C17—H17	119.6
C17—C12—C13	118.10 (12)	C16—C15—C14	119.57 (14)
C17—C12—C11	120.65 (12)	C16—C15—H15	120.2
C13—C12—C11	121.25 (11)	C14—C15—H15	120.2
C7—C8—C9	123.69 (10)	C14—C13—C12	120.87 (13)
C7—C8—C10	118.37 (10)	C14—C13—H13	119.6
C9—C8—C10	117.94 (9)	C12—C13—H13	119.6
C2—C3—C4	121.03 (12)	C6—C5—C4	120.46 (12)
C2—C3—H3	119.5	C6—C5—H5	119.8
C4—C3—H3	119.5	C4—C5—H5	119.8
N1—C9—C8	177.26 (13)	C1—C6—C5	120.71 (13)
C8—C7—C4	131.72 (10)	C1—C6—H6	119.6
C8—C7—H7	114.1	C5—C6—H6	119.6
C4—C7—H7	114.1	C15—C14—C13	120.19 (14)
N2—C11—C12	113.92 (10)	C15—C14—H14	119.9
N2—C11—H11A	108.8	C13—C14—H14	119.9
C12—C11—H11A	108.8	C2—C1—C6	119.82 (13)
N2—C11—H11B	108.8	C2—C1—H1	120.1
C12—C11—H11B	108.8	C6—C1—H1	120.1
H11A—C11—H11B	107.7	C15—C16—C17	120.48 (14)
O1—C10—N2	123.51 (11)	C15—C16—H16	119.8
O1—C10—C8	119.71 (10)	C17—C16—H16	119.8
N2—C10—C8	116.76 (10)		
			
C5—C4—C3—C2	0.8 (2)	C9—C8—C10—N2	8.94 (16)
C7—C4—C3—C2	−179.17 (12)	C4—C3—C2—C1	−0.6 (2)
C7—C8—C9—N1	−153 (3)	C13—C12—C17—C16	−0.1 (2)
C10—C8—C9—N1	27 (3)	C11—C12—C17—C16	179.64 (13)
C9—C8—C7—C4	−1.4 (2)	C17—C12—C13—C14	0.1 (2)
C10—C8—C7—C4	178.30 (11)	C11—C12—C13—C14	−179.67 (12)
C5—C4—C7—C8	−4.2 (2)	C3—C4—C5—C6	−0.9 (2)
C3—C4—C7—C8	175.79 (12)	C7—C4—C5—C6	179.10 (14)
C10—N2—C11—C12	109.92 (13)	C4—C5—C6—C1	0.7 (3)
C17—C12—C11—N2	104.01 (14)	C16—C15—C14—C13	0.0 (3)
C13—C12—C11—N2	−76.26 (15)	C12—C13—C14—C15	0.0 (2)
C11—N2—C10—O1	−7.34 (18)	C3—C2—C1—C6	0.4 (2)
C11—N2—C10—C8	171.11 (10)	C5—C6—C1—C2	−0.5 (3)
C7—C8—C10—O1	7.69 (17)	C14—C15—C16—C17	0.0 (3)
C9—C8—C10—O1	−172.55 (11)	C12—C17—C16—C15	0.1 (3)
C7—C8—C10—N2	−170.82 (11)		

### Hydrogen-bond geometry (Å, °)

**Table:**

*D*—H···*A*	*D*—H	H···*A*	*D*···*A*	*D*—H···*A*
N2—H4···N1^i^	0.88 (1)	2.24 (1)	3.0687 (14)	157.(1)
C3—H3···O1^ii^	0.93	2.36	3.2672 (16)	164

Symmetry codes: (i) −*x*+1, −*y*, −*z*+1; (ii) −*x*, −*y*+1, −*z*+1.
